# Holographic optical field recovery using a regularized untrained deep decoder network

**DOI:** 10.1038/s41598-021-90312-5

**Published:** 2021-05-25

**Authors:** Farhad Niknam, Hamed Qazvini, Hamid Latifi

**Affiliations:** 1 Laser and Plasma Research Institute, Shahid Beheshti University, Tehran, 1983963113 Iran; 2 Department of Physics, Shahid Beheshti University, Tehran, 1983963113 Iran

**Keywords:** Engineering, Mathematics and computing, Imaging and sensing, Optics and photonics, Microscopy, Interference microscopy, Optical imaging, Imaging, Microscopy, Interference microscopy

## Abstract

Image reconstruction using minimal measured information has been a long-standing open problem in many computational imaging approaches, in particular in-line holography. Many solutions are devised based on compressive sensing (CS) techniques with handcrafted image priors or supervised deep neural networks (DNN). However, the limited performance of CS methods due to lack of information about the image priors and the requirement of an enormous amount of per-sample-type training resources for DNNs has posed new challenges over the primary problem. In this study, we propose a single-shot lensless in-line holographic reconstruction method using an untrained deep neural network which is incorporated with a physical image formation algorithm. We demonstrate that by modifying a deep decoder network with simple regularizers, a Gabor hologram can be inversely reconstructed via a minimization process that is constrained by a deep image prior. The outcoming model allows to accurately recover the phase and amplitude images without any training dataset, excess measurements, or specific assumptions about the object’s or the measurement’s characteristics.

## Introduction

### Approach overview and background

Digital holographic microscopy is one of the most widely explored modalities as it permits high-throughput multi-dimensional imaging of phase and amplitude information of the specimen. In the meantime, Gabor-based Lensless In-line Holographic Microscopy (LIHM)^1–6^ has attracted special attention because of its simplicity, compactness, and high space-bandwidth product. In any coherent holographic configuration, it is typical to have coherence-related noises such as speckle, defocus, self/cross-interference artifacts. However, due to incomplete data acquisition by digital image sensors which only contain the intensity information of the complex optical field, the LIHM reconstruction gives rise to images overlaid by a spatial artifact called *twin image.* Although this problem can be robustly solved in off-axis geometry by angled illumination which involves an optical setup^7–10^, this effect can be computationally eliminated by imposing physical constraints that the twin image does not satisfy. Such constraints can be performed through iterative error reduction procedures known as phase retrieval^11–13^.

In any phase retrieval layout, information diversity must be conducted. Then, the problem can be solved using the excess information as a prior which constrains the possible solutions by reducing the number of unknowns. For instance, one can record multiple holograms in different sample-to-sensor distances and recover the complete object information through a physics-based iterative process^6,14,15^. Such multi-holograms can also be retrieved from multiple wavelengths^16^, angles^17^, or phase shifts^18,19^; generally derived from the methods of alternating projections^11–13,20,21^. Despite their robustness, *Multiple measurements* are the backbone of these approaches that limits the usage of LIHM for different imaging problems, especially with poor image acquisition rate. Furthermore, these techniques are susceptible to instrumentation errors or other undesired sample-specific or per measurement of environmental noises.

Another strategy is to inversely estimate the complex-valued object field. By integrating known prior information about the object in the reconstruction process such as object-support^22^ or bound and sparsity constraints^23–26^ in the object’s original or transformed domain, the desired optical properties of the object could be retrievable. However, since such an approach requires handcrafted image priors and perfectly tuned parameters, this strategy is limited to very specific small, simple, or undetailed categories of objects.

In recent years, deep neural networks (DNN) trained in an end-to-end fashion on large datasets have been used for phase retrieval, directly mapping measured intensities to fully resolved object fields. Different studies have demonstrated the state-of-the-art performance of DNNs in various imaging problems^27–31^ such as LIHM^32,33^.

In such techniques, a neural network learns the correspondence between the measured and the ground-truth information through a training process. Then, based on the trained image-to-image mapping features, the network could map the different input measured images to approximations of their ground-truth equivalents. However, for nonconvex nonlinear problems, DNNs would learn specific characteristics of the training data that do not necessarily map to the desired distribution for the experimental data. This is a known issue in all data-driven reconstruction strategies that may lead to failures when predicting patterns that were not provided in the training dataset^34^.

Here we demonstrate a new reconstruction algorithm for LIHM based on a generative untrained deep neural network which is incorporated with a physical model. Inspired by recent successful implementations on several phase retrieval problems^35,36^, this approach is based on the idea of image generation using a randomly initialized DNN; the so-called deep image prior (DIP) devised by Ulyanov et al.^37^. Based on this concept, if a randomly initialized deep network is optimized to fit an image, the convolution layers would act as a natural image prior which enables the network to restore the image from defects such as noises and artifacts.

Using the concept of DIP, in Ref.^35^ the authors demonstrated that an off-axis hologram could be reconstructed by fitting an untrained encoder–decoder network to the hologram through the holographic reconstruction algorithm. Though their implementation is only valid when the absorbance is negligible, and the object field can be solely described in terms of phase. In Ref.^36^, it is demonstrated that not only the DIP can be incorporated with a physical model to recover the phase information of an object, but also it expands the possibilities of involving other complicated constraints e.g., solving problems that are deeply connected to the image formation process such as optical aberrations.

In the context of lensless in-line holographic reconstruction, the proposed approach in^35^ is applicable to transparent phase-only samples. Assuming the amplitude distribution is uniform, the indeterminism in the phase recovery process will be minimized. However, for real-life applications, there are limited choices as phase-only substances. Most tissues and living cells exhibit at least some portion of absorption in the visible spectrum regardless of other optical effects like refraction.

Although the acquired information is not enough to fully resolve the object, as explained in^36^, a DIP model can be versatile enough to accept externally defined priors to overcome the under-sampling problem of in-line holography. These priors could be generic constraints on the network parameters or their outcomes.

For instance, a simple under-parameterized network (has fewer weights than the number of image pixels) is proposed in^38^ called deep decoder network (DDN) which is also exploited in^36^. Thanks to its few parameters, this network naturally performs a regularization by simplifying the representation of the image signal. DDNs are simple, robust, and do not require any early stopping. Another advantage is their stronger regularization imposed by their learned priors on the basis of fewer parameters. This makes them more resilient to noise in optimization problems compared to complicated convolution networks such as encoder-decoders. Furthermore, a DDN is basically initialized by a constant random tensor. This feature of fixed input enables more control over the network parameters and its outputs, which allows performing scheduled variation in inputs and parameters without worrying about any side effects such as chaotic behavior or instability.

The under-parameterization of DDNs -alone- is not enough to confront the indeterminism of the problem. However, by introducing a particular set of constraints on the penalty function, it is possible to modify the DIP of the deep network such that the model could effectively recover the object information.

In this research, we demonstrate that by utilizing simple and commonly available regularization methods, a DDN with a physical image formation algorithm can be upgraded to a powerful compressive signal reconstruction model. The proposed model can robustly recover amplitude and phase information of Gabor holograms with state-of-the-art performance using far fewer samples than required by the Nyquist criterion. For abbreviation, we name our approach Deep Compressed Object Decoder or DCOD.

### Reconstruction theory and principles of image formation

**Figure 1:**
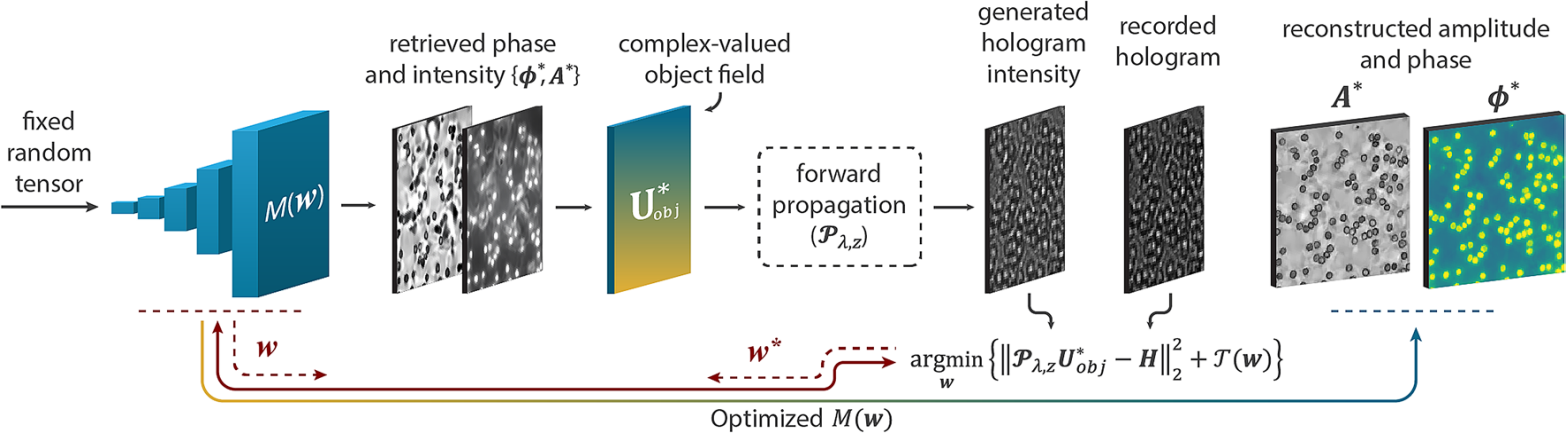
An overview of the proposed reconstruction algorithm. The inverse problem can be solved by optimizing a network of weights by the mean-squared loss between the intensity of the generated hologram and the recorded image in an iterative gradient descent procedure.

In the proposed algorithm (Fig. 1), a randomly initialized DDN (as a universal model) predicts the phase and amplitude images. After constructing a hologram by forward propagation of the estimated object field, the weights of the DDN are updated based on the error between the generated and the recorded holograms in addition to a regularization term. Here, the principles of image formation and the corresponding inverse problem will be described. Following this first step, we will provide a detailed account of the practical implementation.

In general, a thin transparent object at $z=0$ plane can be characterized by a planar complex-valued object function as:

1 $${U}_{obj}\left(x,y;z=0\right)=A\left(x,y\right){\mathrm{exp}}\left(j\phi \left(x,y\right)\right)$$

where $A$ and $\phi$ represent the transmittance and phase responses of the object. By considering an incident coherent wavefront ${U}_{inc}\left(x,y;z=0\right)$ on the object plane, with a wavelength of $\lambda$, at the sensor plane which is placed at a distance of ${z}_{s}$ away from the object, the transmitted and propagated field $U$ can be described using the angular spectrum of the object field on the sensor plane^7^ as:

2 $$U\left(x,y;z={z}_{s}\right)={\mathcal{F}}^{-1}\left\{P({\varvec{\nu}},\lambda ,{z}_{s})\cdot {\mathcal{F}}\left({U}_{inc}\left(x,y;0\right){U}_{obj}\left(x,y;0\right)\right)\right\}$$

where ${\mathcal{F}}^{-1}$ and ${\mathcal{F}}$ are inverse Fourier and Fourier transform operators. $P(.)$ is the free-space optical transfer function that gives a complex-valued matrix and depends on spatial frequency components ${\varvec{\nu}}=({\nu }_{x},{\nu }_{y})$, wavelength $\lambda$, and distance of propagation $z$. $P({\varvec{\nu}},\lambda ,z)$ can be stated as:

3 $$P\left({\varvec{\nu}},\lambda ,z\right)=exp\left(\frac{2\pi jz}{\lambda }\sqrt{1-{(\lambda {\nu }_{x})}^{2}-{\lambda {\nu }_{y}}^{2}}\right)$$

Now assuming the optical field on the sensor plane only includes a diffraction pattern of the object and by considering ${U}_{inc}\left(x,y;0\right){U}_{obj}\left(x,y;0\right)={{\varvec{U}}}_{0}$ in Eq. (2), the intensity of the measured image can become:

4 $$I\left(x,y;z=0\right)={\left|U\left(x,y;z=0\right)\right|}^{2}\equiv {\mathcal{\varvec{P}}}_{\lambda ,z}{{\varvec{U}}}_{0}:{\varvec{H}}$$

${\mathcal{\varvec{P}}}_{\lambda ,z}$ is the propagation operator and ${\varvec{H}}$ is the so-called hologram^7,39–41^.

Equation (4) can be solved by inversely finding an estimate of the object distribution ${{\varvec{U}}}_{obj}^{*}$ for which the corresponding diffraction field ${{\varvec{U}}}^{\boldsymbol{*}}$ has minimum difference with the ground-truth diffraction field ${\varvec{U}}$. This can be carried out using iterative error reduction algorithms such as gradient descent. The DIP approach states that we can make this approximation with a deep network and optimize it by minimizing a penalty function. The idea behind DIP is that the feature extraction process in a randomly initialized untrained convolution neural network forms an optimizable prior that perfectly fits for each reconstruction. Thus, there exists an optimized set of parameters ${\varvec{w}}$ for which the DNN model $M({\varvec{w}})$, can estimate the amplitude ${A}^{*}$ and phase ${\phi }^{*}$ distributions of the object ${{\varvec{U}}}_{obj}^{*}$ whose diffraction intensity at the sensor plane ${\left|{{\varvec{U}}}^{\boldsymbol{*}}\right|}^{2}$ has minimum difference with the recorded hologram ${\varvec{H}}$. This can be formally expressed by a minimization problem with mean squared loss as follows:

5 $$f\left({{\varvec{w}}}^{*}\right)=\underset{{\varvec{w}}}{\mathrm{argmin}}\left\{{\Vert {\mathcal{\varvec{P}}}_{\lambda ,z}{{\varvec{U}}}_{obj}^{*}-{\varvec{H}}\Vert }_{2}^{2}+{\mathcal{T}}({\varvec{w}})\right\}$$

$f\left({{\varvec{w}}}^{*}\right)$ is the minimized loss function that returns the optimal ${{\varvec{w}}}^{*}$ argument for which $M({{\varvec{w}}}^{\boldsymbol{*}})$ gives the reconstructed images. Assuming ${{\varvec{U}}}_{inc}={\mathbf{1}}$ , then ${{\varvec{U}}}^{\boldsymbol{*}}={\mathcal{\varvec{P}}}_{\lambda ,z}{{\varvec{U}}}_{obj}^{*}$ where ${{\varvec{U}}}_{obj}^{*}$ can be expressed as Eq. (1), whereas ${{\varvec{\phi}}}^{*}={M}_{\phi }({\varvec{w}})$ and ${{\varvec{A}}}^{*}={M}_{A}({\varvec{w}})$. ${M}_{\phi }$ and ${M}_{A}$ are the dual output channels of the network $M$. ${\mathcal{T}}({\varvec{w}})$ is an externally defined regularization term. It is clear that the ground-truth object function ${{\varvec{U}}}_{obj}$ did not appear in Eq. (5) and the optimization process is only driven by a single measurement, ${\varvec{H}}$. Note that the periodic nature of the complex exponential phase term in Eq. (1), causes the recovered phase to be limited to the range $[-\pi ,\pi ]$; resolving this limitation is beyond the scope of this study.

For thin and transparent samples, it is possible to encounter the problem as phase retrieval of a phase object. This assumption immediately eliminates the issue of incomplete measurement and allows to solve Eq. (4) without defining any specific regularization. Assuming the amplitude term is mostly uniform and has a small contribution in the object field, it can be approximated as a uniform matrix filled with the average value of the background. Consequently, the model $M({\varvec{w}})$ only gives an estimate of the phase matrix ${{\varvec{\phi}}}^{*}$ while its corresponding amplitude becomes ${{\varvec{A}}}^{*}={\varvec{I}}$. ${\varvec{I}}$ is a constant matrix in which ${{\varvec{I}}}_{{\varvec{i}}{\varvec{j}}}=s\in {\mathbb{R}}$ for all $i,j\in \{0,\dots ,{n}_{d}-1\}$ where $s=mean(background)$. It is based on the PhysenNet framework proposed in^35^ which is implemented on a U-Net, and its minimization does not require the regularization term in Eq. (5).

### Resolving the incomplete signal using a regularized DDN

Since the measured signal ${\varvec{U}}$ is incomplete regardless of the level of noise, Eq. (4) is ill-posed and Eq. (5) becomes hard to solve. The most important issue here is the twin image problem which cannot be suppressed by reducing the spatial details of the object to the features. Such a source of noise imposes a severe level of nonlinearity in Eq. (4) which overlays on the object diffraction field across the entire space and requires carefully defined priors to penalize. Thickness or axial distribution of the object are other issues that add further complication to Eq. (5). Despite the regularization of DDN, without any strongly confining priors on the solutions, the network has the capacity to produce noise. The purpose of the regularization term ${\mathcal{T}}({\varvec{w}})$ in Eq. (5) is to address this problem. This term may be expanded to the following arguments:

1. Assuming the values of the solution are limited to a particular range, one simple prior would be a bound constraint on the solutions. Such constraint can be easily fulfilled by applying a bounded activation function on the output layer such as sigmoid. This function can limit the resulting values of each amplitude and phase solution to [0, 1].
2. Assuming the variables are small, independent, and uncorrelated, energy minimization regularization algorithms such as the ${\ell}_{2}$ regularization or the so-called weight decay reduces (or removes) the contribution of every non-significant component in the features space which substantially improves the reconstruction outcome.
3. Since the loss function is non-convex, minimizing the cost may not necessarily lead to a true solution. Assuming the loss function has a global minimum, perturbing the parameters periodically could prevent overfitting on a particular set of parameters.

About the latter argument, note that our purpose is to fit the model output to a single measured image signal. As a result, overfitting the noise is very probable even with the presence of the powerful DDN image prior and the weight decay in the reconstruction process. Information diversity in supervised neural networks is proved to be an effective way to generalize the trained models and to prevent overfitting. Although it is impossible to define such diversity without any dataset, by applying scheduled random perturbations to the weights, the model tries to optimize the network on a slightly different set of parameters. Random noise could be added directly to the model parameters, to the input tensor of the DDN, or the outputs of the network.

**Figure 2:**
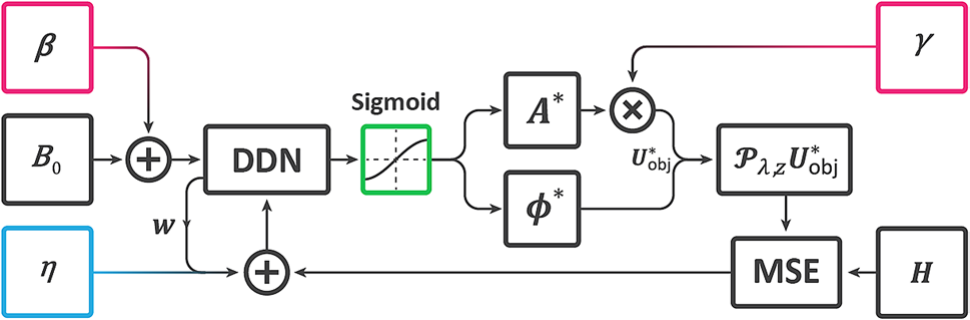
Structure of the DCOD algorithm. Colored boxes are the regularization elements.

Considering $\gamma$ as a positive scalar variable, ${\varvec{\beta}}$ as a tensor variable with the same shape of ${{\varvec{B}}}_{0}$ which is the input tensor of the network, and $\eta$ as the weight decay, the model could be visualized as Fig. 2. $\gamma$ and ${\varvec{\beta}}$ are randomizing variables that could be randomly changed during the optimization to perturb the network parameters.

## Results

**Figure 3:**
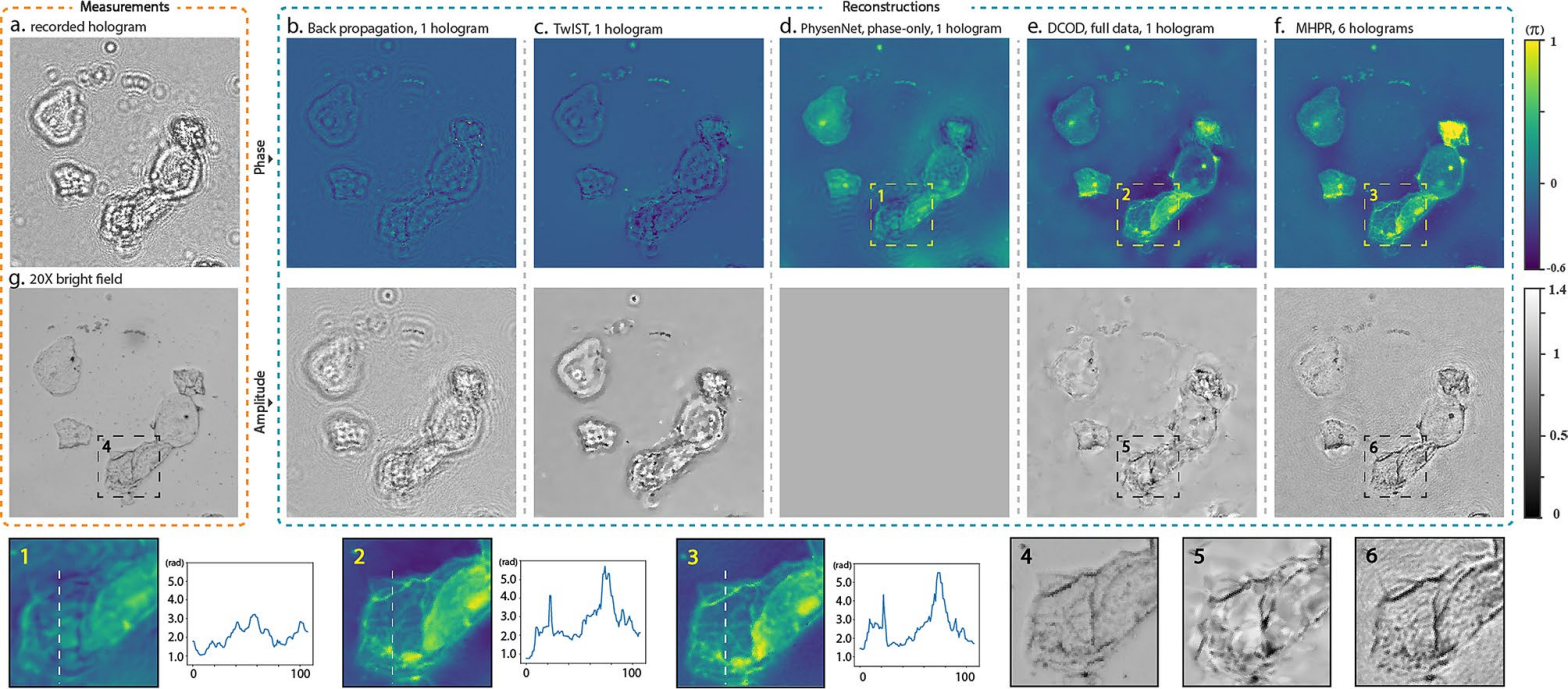
(**a**) The recorded hologram. (**b**) The backpropagated phase and amplitude images. (**c**) The images obtained by TwIST algorithm. (**d**) The reconstructed phase by PhysenNet assuming the object function only contains phase information. (**e**) The recovered phase and amplitude images by the proposed DCOD algorithm. (**f**) The reconstructed images obtained by multi-height phase recovery algorithm. (**g**) The bright-field microscopic image captured using a 20× objective lens (0.4NA).

Figure 3 shows the recorded intensity image (Fig. 3a) and its reconstructed phase and amplitude images of a sample of smeared unstained cheek-cells placed at $238\,\upmu {\mathrm{m}}$ from the sensor plane.

The backpropagation results shown in Fig. 3b are directly solved by propagating the hologram to the object plane, or ${{\varvec{U}}}_{rec}={\mathcal{\varvec{P}}}_{\lambda ,-z}{\varvec{H}}$. The reconstructed object information in ${{\varvec{U}}}_{rec}$ is disturbed and highly entangled in the overlaying intense twin image noise which is the result of incomplete data acquisition. In summary, Fig. 3b is a visualization of the fundamental ill-posedness of the problem.

Figure 3c depicts the results obtained by TwIST (Two-step Iterative Shrinkage/Thresholding^42^) algorithm accompanied with a TV-regularizer (Supplementary section A). This approach is widely used to compressively solve inverse problems as holographic reconstruction^24,25,43^. Due to the small sample-to-sensor distance of the adopted LIHM experimental setup, the field information is encoded within intensely entangled interferometric patterns from which, TwIST algorithm cannot properly reconstruct complex geometries. In fact, the outcomes are merely TV-denoised versions of the backpropagated images.

Figure 3d is obtained by a PhysenNet which is made on top of a DDN as the decoder unit and is initialized with a random tensor. The deep network is followed by the computational propagation of the generated object field creating the output hologram. Assuming the amplitude of the object is uniform, the network-made phase image is combined with a uniform matrix as the amplitude to form the complex-valued object function [Eq. (1)]. The problem is then solved using the Adam optimization algorithm^44^, which minimizes the Mean Squared Error (MSE) of the generated holograms. Finally, optimization is performed with a learning rate of 0.01 for 20,000 iterations. The phase-only constraint, although simple and effective, allows little flexibility against any model mismatch imposed by amplitude variations and thus, fails to resolve the object phase.

Figure 3e illustrates the reconstructed images obtained by the proposed algorithm and the object field is fully recovered using only a single hologram. The outcomes are comparable with the images obtained by the Multi-Height Phase Recovery method (MHPR, Fig. 3f) and imply that the proposed model outperforms TwIST. The optimization of the DCOD is carried out using a weight decay-included Adam optimizer called AdamW^45^ with a learning rate of 0.01 and a weight decay of 0.002 for 35,000 iterations. The results obtained by this algorithm have excellent agreement with the images acquired by MHPR (using 6 axially spaced holograms) and bright field microscopy (using a 20× objective lens, Fig. 3g). The regularization steps taken to overcome the nonlinearity are discussed thoroughly in the subsequent section.

It is worth mentioning that the model clearly failed to recover the bright spot in Fig. 3f that has a large phase value $(>\pi )$. In such circumstances, the optimizer gets confused since we did not define any mechanism to perform phase unwrapping.

## Discussion

### Regularization techniques

Since the inverse problem in in-line holography is inherently nonconvex and nonlinear, besides applying positivity and bound constraints on the solutions, it is crucial to penalize complexity by performing further regularizations to help the model converge.

We applied different techniques to improve convergence. One common way is to contribute the weights in the loss function multiplied by a coefficient called weight decay^46^ to encourage the weights to become small. For Stochastic Gradient Decent (SGD) algorithm, weight decay regularization is equivalent to ${\ell}_{2}$ regularization of the loss function. This can be formally expressed regarding $\frac{\eta }{2\alpha }{\Vert {\varvec{w}}\Vert }_{2}^{2}$ as the regularization term ${\mathcal{T}}({\varvec{w}})$ in Eq. (5), where $\eta$ and $\alpha$ are weight decay and learning rate parameters respectively. As stated in^45^, For adaptive gradient-based methods (Adam^44^, AdaGrad^47^, AMSGrad^48^, etc.), this resemblance is not valid since with ${\ell}_{2}$ regularization, the regularizer takes the sums of the gradient of the loss function and the gradient of the regularizer into account, while with weight decay regularization, only the gradient of the loss function needs to be adopted with the regularizer. Therefore, a modified variant of Adam optimizer called AdamW is proposed that decouples loss-based gradient updates in Adam and weight decay, which substantially improves the regularization efficiency^45^. It is demonstrated that decoupled weight decay regularization not only provides flexibility for hyperparameter tuning, but also improves generalization for different experimental settings.

In order to further improve convergence, regularization is extended by introducing randomness to the model parameters. We applied randomization in two ways: first, by adding random noise to the initial fixed random tensor, and second, by multiplying a periodically varying coefficient $(>1)$ to the generated intensity image, which effectively perturbs the parameters of the network. Periodic random perturbations enable the neural network to give more emphasis to robust features across detailed environments. We observed that applying random perturbations to the model parameters significantly accelerates convergence and gives rise to reconstructions with more meaningful information.

**Figure 4:**
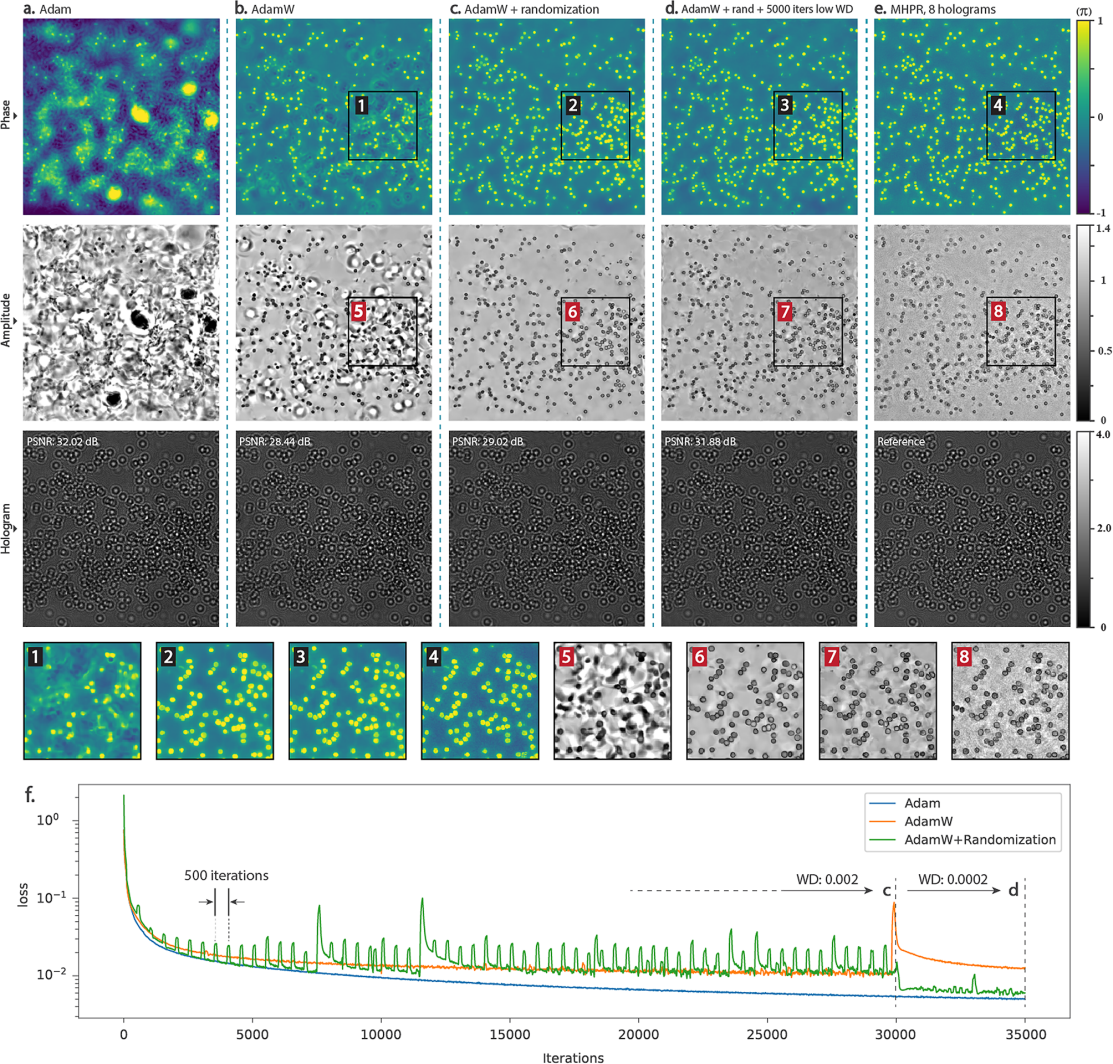
Reconstructed phase, amplitude, and diffraction (at the sensor plane) images of smeared red blood cells with different regularization settings. The results are obtained after 30,000 iterations (**a**) without any regularization, (**b**) with a weight decay of 0.002, (**c**) with randomizations applied every 500 iterations, and (**d**) with the same settings of (**c**) with additional 5000 iterations through which, the weight decay is 0.0002 (no randomization). (**e**) The images obtained by the MHPR method using 8 holograms. (**f**) MSE of the generated holograms. Spikes on the green curve are caused by random perturbations.

To investigate the influence of these modifications, another experiment is conducted on smeared red blood cells; the results of which are shown in Fig. 4. As depicted in Fig. 4a, the model failed to find the solution with unsophisticated implementation of DDN with Adam optimizer. In Fig. 4b as well, due to lack of randomization, the true solution is lost across a broad range of possible solutions giving similar diffraction intensities on the hologram plane. Unlike the previous cases, the images generated by our algorithm in Fig. 4c,d have clear equivalence with the images obtained by the MHPR method (Fig. 4e). Specifically, the images in Fig. 4c are general representations of phase and amplitude that are regularized by randomization which was applied by perturbing the network every 500 iterations for 30,000 iterations. The initial output is over smoothed, hence with 5000 more iterations using a lower weight decay (0.1 of the initial value) and without randomization, more details will appear (Fig. 4d).

Figure 4f shows MSE variations during the optimization for different regularization settings mentioned. The spike that appeared on about 30,000 iterations on the AdamW graph (orange curve) is evidence of instability. Due to the lack of scheduled reduction of weight decay and learning rate during training, AdamW becomes unstable after several thousand iterations and requires early stopping. This instability is more obvious after letting the optimization run up to 100,000 iterations (see Supplementary Fig. S1). Another benefit of randomization is to stabilize the optimization when weight decay regularization is applied.

The generated holograms at the sensor plane shown in Fig. 4 are hardly distinguishable. This is the result of overfitting when regularization is not applied appropriately. Furthermore, the PSNR and MSE values are not showing any meaningful differences between the holograms and the object images. This effect is another consequence of overfitting.

## Limitations

The problem of inverse reconstruction of an in-line hologram is underdetermined by (at least) a factor of 2; a real-valued intensity image against a complex-valued object function. Compressive sensing techniques address this problem by reducing the number of required parameters, assuming numerical constraints, and enforcing prior information about the object in the reconstruction process. For this reduction, it is required to transform the problem from the pixels space to the features space. Similarly, the DDN builds a pixel-wise estimate of the object field by a linear combination of feature maps. Hence, the performance of the reconstruction is limited by the level of indeterminism of the problem which is directly related to the amount of spatial (or spectral) details of the object, and the amount of optical information required to resolve through the reconstruction. These are two major limiting factors constraining the performance of the proposed method. The influences of these limitations will be explained by the following two experiments.

### Level of ill-posedness

To investigate the effect of ill-posedness on the reconstruction performance, two general examples were considered in which, the effects of strength and distribution of details are examined. For both cases, five holograms were computationally generated by two randomly selected images as phase and amplitude.

In the first example, the amplitude image for each hologram is blurred by a Gaussian function with different degrees of smoothing power. The blurriness degree which is defined by a standard deviation parameter ($\sigma$) increases exponentially for each image such that $\sigma$: $exp(0)$, $exp(1)$, …, $exp(3)$. The dimensions of the Gaussian kernels are defined by $\sigma$ such that $d\left(\sigma \right)=6\times [\sigma ]+1$ where [.] is the round operation. This example gives us an insight into the influence of the distribution of spectral components on the ill-posedness of the problem and therefore, the accuracy of the reconstructions.

In the second example, five amplitude images with different contrasts are considered. The contrasts have a descending order from 100 to 0% with a 25% increment. This experiment is designed to investigate the relation between the power of spectral components of the object and the difficulty of the problem.

**Figure 5:**
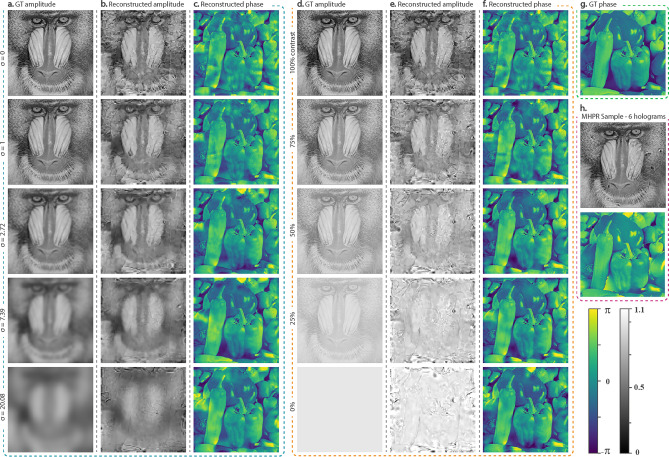
(**a**) Ground-truth (GT) amplitude images blurred by a Gaussian function with different degrees of standard deviation. (**d**) GT amplitude images with different contrasts. (**b**, **c**, **e**, **f**) Reconstructed images of (**a**, **d**, **g**) as the amplitude and phase images. (**h**) Reconstructed images achieved by MHPR method using 6 axially shifted holograms.

The input images and their reconstructions for both cases are shown in Fig. 5. The phase images in both experiments are unchanged for comparison.

The reconstructed images in Fig. 5 show that with any reduction of diversity and strength in details, the accuracy of the reconstructions improves. One explanation is that the Gaussian blur smooths the sharp edges of the amplitude image, which normally is the cause of ringing artifact; this effect is posed by the band limit in discrete Fourier analysis and is much more visible when phase and amplitude images are not geometrically correlated. Secondly, in some areas of the amplitude image, the pixel values are close to zero and because of finite sampling of digital image sensors, the phase information of those areas gets scrambled. Theoretically, these explanations are valid but as illustrated in Fig. 5h, these issues have minimum influence on the reconstructions when the available information about the object is enough.

To achieve a quantitative understanding of the results, we need to introduce several indices. The Normalized Root Mean Square (NRMS) index can be regarded as a measure of the scattering power of the holograms. NRMS of each hologram intensity image ${\varvec{H}}$ can be calculated as:

6 $$NRMS({\varvec{H}})=\frac{RMS({\varvec{H}}-{\varvec{r}}{\varvec{e}}{\varvec{f}})}{RMS({\varvec{r}}{\varvec{e}}{\varvec{f}})}$$

where ${\varvec{r}}{\varvec{e}}{\varvec{f}}$ is the reference (or background) matrix. Moreover, the power spectral entropy (PSE)^49^ of an image shows the information’s uncertainty in a given spectrum which can be regarded as a measure of complexity. PSE of each amplitude image, ${\varvec{I}}$, can be calculated as:

7 $$PSE\left({\varvec{I}}\right)=-\sum_{i=1}^{n}{p}_{i}{\mathrm{log}}_{2}{p}_{i}$$

where ${p}_{i}$ is the normalized PSE for each frequency component ${\omega }_{i}$ and can be described as:

8 $${p}_{i}=p\left({\omega }_{i}\right)=\frac{P({\omega }_{i})}{\sum_{i}P({\omega }_{i})}$$

$P$ is the PSE of the image ${\varvec{I}}$. Furthermore, the visual similarity between the reconstructed images and their ground-truth equivalents is commonly evaluated using the Structural Similarity Index Measure (SSIM). This quantitative measure of quality could be regarded as a measure of ill-posedness.

For both examples, these indices are calculated and arranged in Tables 1 and 2. For the first case, the NRMS values of the hologram images are showing that the information diversity in each amplitude image does not necessarily affect the scattering power of the hologram. The PSE values of the input amplitude images are showing a reduction of complexity with any increase of blurriness.

**Table 1:** Comparison of the SSIM and PSE indices of the reconstructed images in Fig. 5b,c respect to the scattering power of each simulated hologram.

Gaussian kernel STD*: $\sigma$	0	1	2.72	7.39	20.08
NRMS	0.71	0.71	0.72	0.72	0.71
PSE ($\times {10}^{3}$)	4.0914	3.1272	2.0614	1.6437	1.4758
**SSIM**					
Amplitude	0.30	0.43	0.58	0.69	0.60
Phase	0.57	0.65	0.69	0.72	0.69

*STD standard deviation.

**Table 2:** Comparison of the SSIM and PSE indices of the reconstructed images in Fig. 5e,f respect to the scattering power of each simulated hologram.

Contrast	100%	75%	50%	25%	0%
NRMS	0.71	0.63	0.55	0.52	0.59
PSE ($\times {10}^{3}$)	4.0914	4.0915	4.0912	4.0923	–
**SSIM**					
Amplitude	0.30	0.26	0.19	0.11	0.65
Phase	0.57	0.65	0.71	0.72	0.71

For the images in Fig. 5d, the NRMS of the simulated holograms have a descending behavior while the PSE of the amplitude images (except for zero contrast) is approximately constant. However, this opposite behavior of indices is followed by a descending behavior of the ill-posedness.

According to the SSIM values denoted in Table 1, information reduction directly results in improved reconstruction quality both in amplitude and phase. But for the case of contrast variation, another effect takes place as well. The SSIM values for the amplitude images denoted in Table 2 are implying the more obscured the details are, the more intensely the model filters them out, although the frequency components are the same. In other words, low-intensity details are regarded as noise and are more likely to be eliminated as can be seen in Fig. 5d. This behavior could be a result of shrinkage regularization of weight decay which inclines the model to an underfitting situation. The opposite slopes of variations in the SSIM values in Table 2 is a quantitative appearance of this behavior.

Regarding the scattering power of the hologram or complexity of the amplitude in each image set, the level of ill-posedness of the problem controls the performance of the reconstruction. This level increases when the specimen is thick or has any axial distribution. Hence, it is not surprising to say that the model performs better when resolving sparsely distributed details in a smooth and well-illuminated background which is the case of thin microscopic biological samples.

### Sparsity-fidelity tradeoff

**Figure 6:**
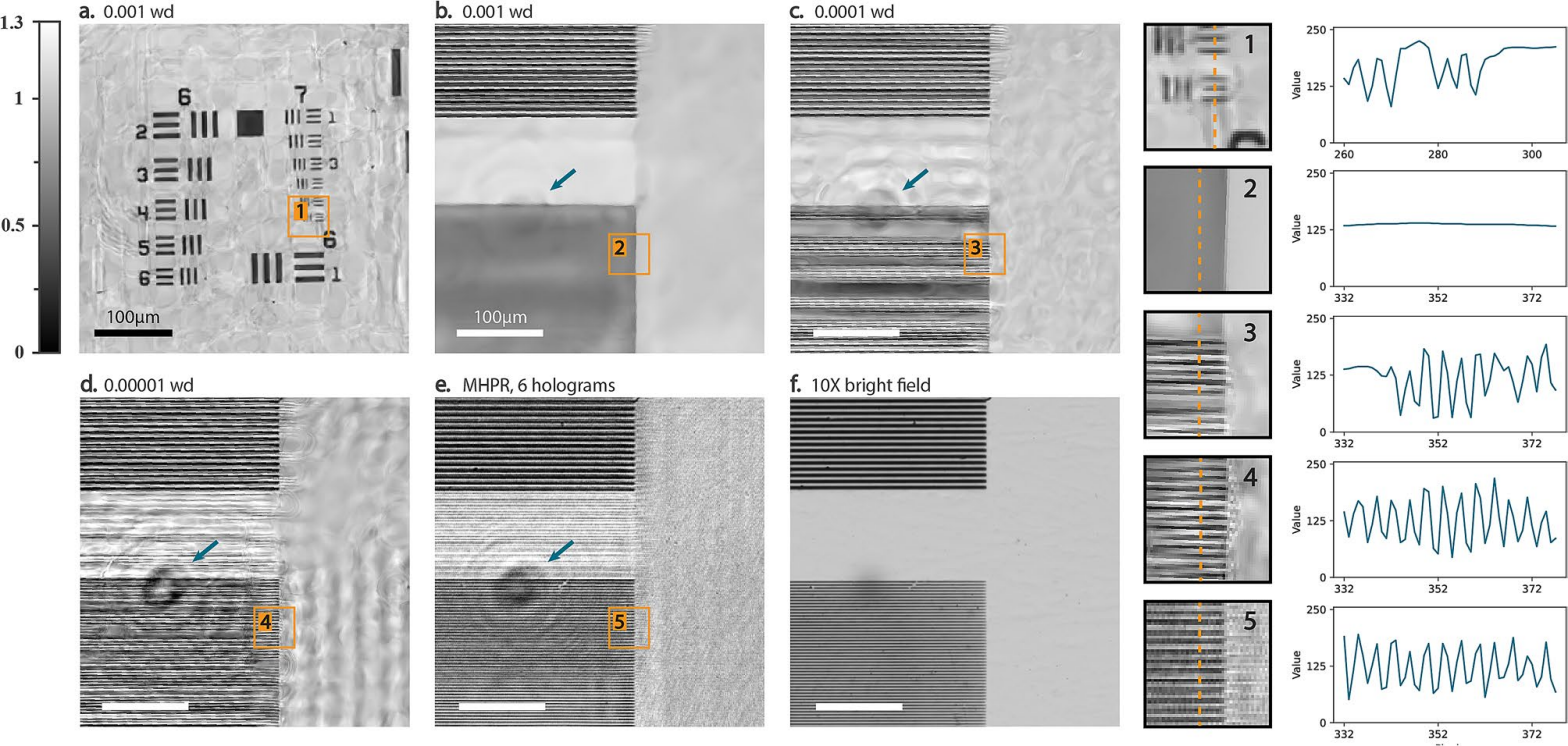
(**a**) the reconstructed amplitude of a standard resolution target (228 lp/mm for the finest details). (**b**, **c**, **d**) The amplitude images recovered from the transmissive grating (125 and 250 lp/mm) by DCOD with weight decay ranging from ${10}^{-3}$ to ${10}^{-5}$. (**e**) The reconstructed amplitude image obtained by multi-height phase recovery method using 6 holograms. (**f**) The microscopic image captured from the same field of view using a 10× 0.2 N.A. objective lens.

When the object function is seen as a set of features, the resolving power of the model would depend on the geometrical characteristics of the details. When regularization and under-parameterization are applied simultaneously, it is unlikely to achieve the maximum resolution for every geometrical feature. To visualize this relationship between the geometrical characteristics of the object and the resolving power of the model, two types of samples are provided: a USAF 1951 standard resolution target, and a transmissive grating. The reconstructed amplitude images of these samples are shown in Fig. 6. The grating has 125 and 250 line pairs per millimeter while the finest section in the resolution target has 228 lp/mm which approximately has the same linewidth of ~ 2 µm.

The finest details of the resolution sample in Fig. 6a are accurately resolved while -with the same model settings- the fine lines in the grating sample are averaged as a smooth area. Fine details in both samples require the same resolving power but the model prioritizes small, isolated features and ignores pattern-like features as noise. As illustrated in Fig. 6c,d, the fine grating pattern can be recovered by decreasing the weight decay, but it comes with the expense of amplified noise.

Different phenomena affect the resolution of the reconstructions e.g., SNR (signal-to-noise ratio), or information diversity i.e., the amount of information acquired under different experimental conditions. The information diversity for our experiments is constant but the under-sampling causes ill-posedness as examined in the previous section. Furthermore, with sufficient exposure to illumination, the contribution of environmental noises tends to zero while the coherent-related noises persistently keep their contribution in the signal. While these issues are consistent in the problem, the fine details could be resolved with lower regularization ratios as shown in Fig. 6c,d. Hence, the smoothing effect in Fig. 6b cannot be related to the noises or information diversity. This effect is a consequence of a fundamental tradeoff between sparsity and fidelity; the fine lines in the resolution sample are distributed much sparser than in the grating.

Weight decay regularization does not impose sparsity but reduces the variations of the features obtained from the object. The variation reduction occurs in the parameter space under the influence of the under-parameterization of the network which results in the extraction of a simplified representation of the object in the feature domain. Weight decay further penalizes the parameters to gather as few varying features as possible, thus enforces a sparsity constraint on the feature extraction process. Sparsity promotion in the proposed method manifests as a smoothing effect over large spatial features and its strength can be controlled by the weight decay coefficient. In general terms, this effect improves the overall quality of the reconstructions by removing high-frequency noises especially the artifacts originating from other sources than the object plane e.g., the fringes formed by an out-of-focus object marked on the images in Fig. 6.

### Outlook

In this study, we demonstrated that an untrained regularized convolution neural network can iteratively recover the object information that is partially available in an in-line hologram. Unlike other methods, this operation does not need to be trained on any dataset or to be supervised by any excess information about the object. The regularization induced by DIP, under-parameterization, weight decay, and randomization that are employed through our demonstration is general and does not need any specific tuning per specimen or external supervision. However, all these advantages come with the price of requiring extensive computational resources and long processing times. Additionally, the trade-off between the resolving power and the noise seems to be fundamental and the problem of limited phase range due to lack of any phase recovery constraint has remained unsolved.

Since the twin image noise typically scrambles phase information, the commonly used deterministic phase unwrapping techniques are usually not helpful in in-line holography. Also, the proposed model fails to reconstruct the wrapped phase correctly and presents random artifacts instead. To overcome this issue, one straight forward way is performing more measurements. With just one more measurement under a different condition, we can obtain an unwrapped estimate of the phase. For instance, if the transport-of-intensity equation (TIE) is applied to several images taken from different heights, it provides an initial phase guess^6^. After that, a regularization term can be defined which will favor achieving a minimized smooth difference between the output phase and the guess. Another alternative idea is using a supervised model to give an unwrapped initial guess of the phase profile. After feeding the results to a fidelity term, the model can be regularized to reconstruct unwrapped phase information. Another possible solution is obtaining multiple phase images through multiple channels with uniquely designed constraints each of which constructing a portion of phase information.

Speed issues can be solved by tuning hyperparameters, adopting an optimized network architecture, or making use of advanced algorithms for randomization, such as warm restart, for greater efficiency. Initializing network parameters (especially the input random tensor) with an encoder unit in a supervised or unsupervised manner might be beneficial as well.

We believe the DCOD structure is versatile enough to be modified to solve other inverse problems with little information about the signal or limited experimental resources such as tomographic imaging using holographic or cross-sectional images, or quantitative phase imaging using a set of intensity images.

## Materials and methods

### Experimental scheme

**Figure 7:**
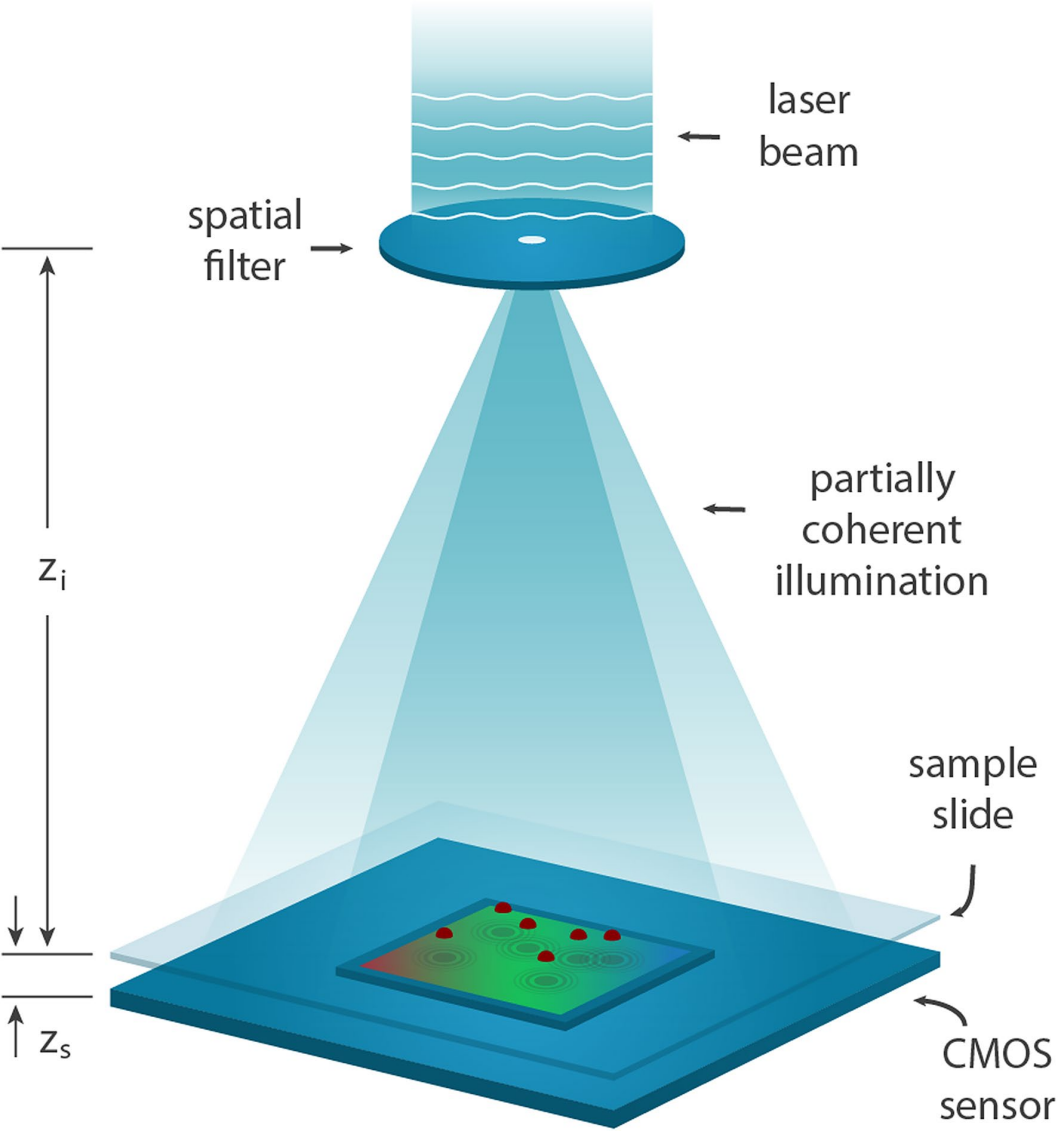
Sketch of the experimental setup for ${z}_{i}\simeq 10\,{\mathrm{cm}}$ and ${z}_{s}<2\,{\mathrm{mm}}$.

A lensless in-line holographic microscope is designed and arranged as schematically shown in Fig. 7. The image sensor is a CMOS Sony IMX-219 which is driven by a Raspberry Pi version 2 camera module. This RGB image sensor provides an array of 10-bit depth (16-bit digital array) $3296\times 2480$ pixels per channel with $1.12\,\upmu {\mathrm{m}}$ pixel pitch which gives an effective area of $3692\,\upmu {\mathrm{m}}\times 2778\,\upmu {\mathrm{m}}$ as the field of view for in-line holographic imaging. A Raspberry Pi 3 B mini-computer board controls the camera system to capture, preprocess, store, and stream the images.

The illumination source includes a 40 mW (max) 532.3 nm Nd:YAG laser with ~ 1 nm bandwidth. The operational power of the laser is reduced to less than 10% for practical use. The beam is spatially filtered by passing through a $20\,\upmu {\mathrm{m}}$ pinhole. The pinhole is also placed $\sim 10\,{\mathrm{cm}}$ away from the sample plane which is far enough to ensure the illumination wavefront is planar compared to the microscopic samples of size $<1000\,\upmu {\mathrm{m}}$. A $3.2\,{\mathrm{cm}}$ focal length lens is placed before the spatial filter to provide a broader thus more uniform illumination on the sensor plane. The sample slide is placed near the sensor plane ($200\,\upmu{\text{m}}{-}2\,{\mathrm{mm}}$) to minimize the effect of undesired cross-interference. Moreover, acquiring additional images at different heights for MHPR could pose undesired lateral displacement against any small variations of height. For small sample-to-sensor distances, this effect could be minimized. With these settings, the illumination light has temporal coherence for a length of $\sim 283\,\upmu {\mathrm{m}}$ (in a vacuum) and spatial coherence for a radius of $\sim 847\,\upmu {\mathrm{m}}$ on the sample plane which is more than enough for typical microscopic samples. This feature of small *sample-to-sensor* $({z}_{s})$/*sample-to-source* $({z}_{i})$ ratio allows achieving unit fringe magnification with a resolution as small as the pixel size of the sensor (if $>\uplambda /2$) and with a field of view as large as the area of the sensor, resulting in a large spatial-bandwidth product.

#### Image acquisition

For each experiment, 6 holograms are captured from a sequence of heights respectively (with $\sim 100\,\upmu {\mathrm{m}}$ interval) due to validation via the MHPR algorithm (Supplementary section B). The hologram with the smallest sample-to-sensor distance is considered as the reference hologram to be adopted by our approach.

#### Preprocessing

The initially recorded raw 10bit Bayer image is needed to demosaic at the beginning. Then, the green channel of the outcoming RGB image is assigned as the hologram. A $512\times 512$ pixels region of each hologram is cropped to feed the model for processing.

To bring quantitative consistency to the measurements, each recorded image is divided with a pre-acquired background image captured in a similar experimental setting.

### Network design

For network *M*, a DDN is designed to map a stack of ${k}_{0}$ many ${n}_{0}$-dimensional random matrices as a tensor ${{\varvec{B}}}_{0}\in {\mathbb{R}}^{{n}_{0}\times {k}_{0}}$, to a ${n}_{d}$-dimensional double-channeled (${k}_{out}=2$) image. In each layer, the transformation process includes an element-wise linear combination of the channels multiplied by a weight tensor followed by upsampling, normalization, and regularization by a rectified linear unit (ReLU). We can write the operations in each given $(i+1)\text{-th}$ layer as:

9 $${{\varvec{B}}}_{i+1}=cn\left(relu({u}_{i}{{\varvec{B}}}_{i}{{\varvec{w}}}_{i})\right) ,\quad i=0,\dots ,d-1$$

${{\varvec{w}}}_{i}\in {\mathbb{R}}^{{k}_{i}\times {k}_{i+1}}$ is the weight tensor of each layer and $u_{i}$ is a bi-linear upsampling operator. $cn\left( \cdot \right)$ performs a channel normalization operation (known as batch normalization) that specifically for its input tensor ${{\varvec{Z}}}_{i}=relu({u}_{i}{{\varvec{B}}}_{i}{{\varvec{w}}}_{i})$, for each channel j, gives:

10 $${{\varvec{Z}}}_{ij}^{^{\prime}}=\frac{{{\varvec{Z}}}_{ij}-mean({{\varvec{Z}}}_{ij})}{std({{\varvec{Z}}}_{ij})}{\gamma }_{ij}+{\beta }_{ij}$$

where $mean$ and $std$ are empirical mean and standard deviation and ${\gamma }_{ij}$ and ${\beta }_{ij}$ are learnable parameters^38^. Subsequently, the output of the *d*-layer network would be:

11 $$\left\{{{\varvec{\phi}}}^{*},{{\varvec{A}}}^{\boldsymbol{*}}\right\}=2\pi sigmoiod\left({{\varvec{B}}}_{d}{{\varvec{w}}}_{d}\right) ,\quad {{\varvec{w}}}_{d}\in {\mathbb{R}}^{{k}_{d}\times 2}$$

which models amplitude ${{\varvec{A}}}^{\boldsymbol{*}}$ and phase ${{\varvec{\phi}}}^{*}$ images through two output channels of M. Based on the layout proposed in^38^, the default network is constructed by 5 similar layers with 256 channels, twofold bi-linear upsampling layers, and an output layer with the same setting but without upsampling. In each layer, a $1\times 1$ convolution operation performs both weight tensor multiplication and linear combination operations as expressed in Eq. (11). The network is finally fed by a random tensor (${{\varvec{B}}}_{0}$) with ${n}_{0}=16\times 16$ and ${k}_{0}=256$ which results in a two channeled ${n}_{d}=512\times 512$ phase-intensity pair tensor. For more details about this network structure, see Supplementary information section D. This framework is implemented using the TensorFlow version 2.3.0 platform in Python 3.6.9.

#### Training

The fixed $16\times 16\times 256$ input tensor contains normally distributed random values with 0 mean and 0.1 standard deviation. In all experiments (except those mentioned particularly in their descriptions), the models are trained with a learning rate of 0.01 and weight decay of 0.002. For weight decay regularization, the TensorFlow implementation of AdamW is adopted from.

With fixed hyperparameters, (for most samples) the proposed model needs about 30,000 iterations to produce satisfactory results which on an Nvidia Tesla k80 GPU, the process takes ~ 40 min.

#### Randomization

Every 500 iterations:

1. A Gaussian noise tensor with a mean of 0 and a standard deviation of 0.02 should be added to the input tensor.
2. The amplitude image should be multiplied by a coefficient whose value switches between 1.3 and 1.4 in every randomization step.

Regardless of randomization, the amplitude image must be multiplied by a coefficient larger than 1, although the amplitude of every pixel is expected to be less than 1 unless the model cannot generate satisfactory results. In general, choosing every value for this coefficient between 1.1 and 1.8 does not show any clear impact on the reconstruction performance.

#### Simulations

For the simulations shown in Fig. 5, the object plane is considered at $z=300\,\upmu{\text{m}}$, the illumination is assumed to be coherent, planar, and has a wavelength of 532.2 nm. Additionally, the pixels of the holograms are assumed to have a size of $1.12\,\upmu{\text{m}}$. For the MHPR method, the height differences are similar and equal to $50\,\upmu {\text{m}}$. The planes are considered further away from the reference plane which is at $z=300\,\upmu{\text{m}}$.

#### Samples

1. Unlabeled de-identified and existing dry Oral epithelial smear slides (cheek cells) and Blood smear slides are obtained from the Microfluidics Laboratory at SBU Laser and Plasma Research Institute.
2. The 1951 USAF standard resolution target is purchased from Thorlabs (# R3L3S1P).
3. The Transmissive grating is fabricated by photolithography technique applied on a copper-coated glass substrate and is obtained from the Surface and Material Laboratory at SBU.

## Supplementary information

Supplementary Information.

## Data Availability

The images and codes are publicly available in https://github.com/farhadnkm/DCOD.
