# Supplementary material for: Holographic optical field recovery using a regularized untrained deep decoder network
Source: Sci Rep. 2021 May 25;11:10903. doi: 10.1038/s41598-021-90312-5 (PMC8149647; doi:10.1038/s41598-021-90312-5)
Supplement: Supplementary file 1 — Supplementary Information. [file 41598_2021_90312_MOESM1_ESM.docx]

***Supplementary Information***

Holographic Optical Field Recovery Using a Regularized Untrained Deep Decoder Network

Farhad Niknam, Hamed Ghazvini, Hamid Latifi

A. TwIST Algorithm

TwIST algorithm solves the regularization problem using the minimizer $f:\mathcal{X}\mathbb{\to R:(-\infty, +\infty)}$ to find the target complex-valued object function $\boldsymbol{x}\in\mathcal{X:}\mathbb{C}^{2}$:

|  | $f\left( \boldsymbol{x} \right)=\underset{\boldsymbol{x}}{\mathrm{argmin}} \left\{ \frac{1}{2}\left\Vert\mathcal{P}_{\lambda,z}\boldsymbol{x}-\boldsymbol{H} \right\Vert_{2}^{2}+\tau TV(\boldsymbol{x}) \right\}$ |  |
| --- | --- | --- |

where $H$ is the measured hologram, $\mathcal{P}_{\lambda,z}$ is the propagation operator, and $\mathcal{P}_{\lambda,z}\boldsymbol{x}$ is the estimated hologram intensity on the sensor plane. $TV(\boldsymbol{x})$ is the total variation regularization of $\boldsymbol{x}$ and $\tau\in[0,+\infty)$ is the regularization parameter. The so-called *isotropic* $TV$ formulation for discrete $TV$ regularization is given by:

|  | $TV\left( \boldsymbol{x} \right)={TV}_{i}\left( \boldsymbol{x} \right)=\sum_{i} \sqrt{\left( \Delta_{i}^{h}\boldsymbol{x} \right)^{2}+\left( \Delta_{i}^{v}\boldsymbol{x} \right)^{2}}$ |  |
| --- | --- | --- |

where $\Delta_{i}^{h}$ and $\Delta_{i}^{v}$ denote horizontal and vertical first-order local difference operators respectively. In every step, a total variation denoising operation is applied which is defined as the denoising function $\psi_{\lambda}:\mathcal{X}\to\mathcal{X}$ that minimizes the Rodin-Osher-Fatemi (ROF) model for a given noisy image $f$ and a regularization parameter $\eta$:

|  | $\psi_{\lambda}\left( \boldsymbol{x} \right)=\underset{\boldsymbol{x}}{\mathrm{argmin}} \left\{ \frac{\eta}{2}\left\Vert\boldsymbol{f}-\boldsymbol{x} \right\Vert_{2}^{2}+TV(\boldsymbol{x}) \right\}$ |  |
| --- | --- | --- |

With $\tau=0.3$and $\lambda=6.0$, this algorithm gives more clarity for our digital holograms.

B. Multi-Height Phase Recovery Algorithm

To achieve the ground truth phase and amplitude images, 6 holograms are acquired and processed using the multi-height phase recovery method. For each hologram, the sample plane is shifted by a positive random increment ($\sim100\mu m$), then the holograms are sorted by height and the smallest height is considered as the first. After finding the z coordinate of each object plane, to extract the object features, the inline reconstructed intensity images are first converted to a binary map by hard thresholding, then registered using a feature matching algorithm [1]. This method is not universal (such as control point mapping), but is effective for sparsely detailed samples.

At the first step, a complex field is made by considering the square root of the first hologram as the amplitude and a zero matrix as the phase. Besides using a zero matrix, the phase matrix can be estimated using transport of intensity equation method which deterministically gives an estimate of the phase distribution. But, since the considered field of view is small in size, the operation is fast enough to eliminate any necessity to employ an advanced algorithm giving a better initial guess.

The created complex field is then computationally propagated to the second hologram plane. Then, the propagated amplitude image and the square root of the second hologram are averaged; together with the propagated phase which is kept unchanged, forms the second complex field. This procedure is repeated all the way to the final plane and then reverse direction until reaching the first plane. This defines one iteration of the algorithm. Usually, after about 100 iterations the results are satisfactory.

C. Optimization in The Long Run


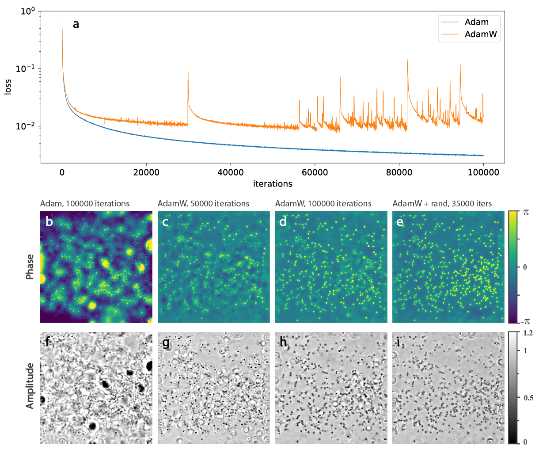


**Figure S1.** **a** variations of the loss value when the reconstruction is performed **b, f** without any regularization (Adam) and **c, d, g, h** with weight decay regularization but without randomization (AdamW). **e, i** reconstructed images with weight decay regularization and randomization.

We cannot claim that the optimization is impossible using solely the AdamW optimizer without randomization. But we observed the optimization performance degrades after several thousand iterations and the further the training proceeds, the more unstable the optimization gets. Figure S1 shows the results generated without randomization after 50000 and 100000 iterations. The Adam optimizer never yields the correct results as depicted in Fig.S1b and Fig.S1f.

D. Network Architecture

The proposed network with similar layers is a default model and changing the structure for different needs is beneficial. To choose a network architecture to replicate this work, the following factors should be notified:

1. The number of parameters relative to the number of output data points which in this case is the number of image pixels multiplied by the number of their channels.
2. The level of details required to resolve through the reconstruction.
3. The computational and memory resources available.
4. The level of noise and complexity of the problem.

For this research, our goal was to obtain maximum clarity with the minimum number of parameters to decrease the computational costs of the process. For an output tensor with a shape of $512 \times512 \times2$, having fewer parameters than 524,288 is sufficient to keep the network under-parameterized. This reduces the effect of over-fitting in the long run which is the whole point of deep decoder networks. Thus, depending on the difficulty of the problem, the network architecture can be adjusted to meet the desired requirements.


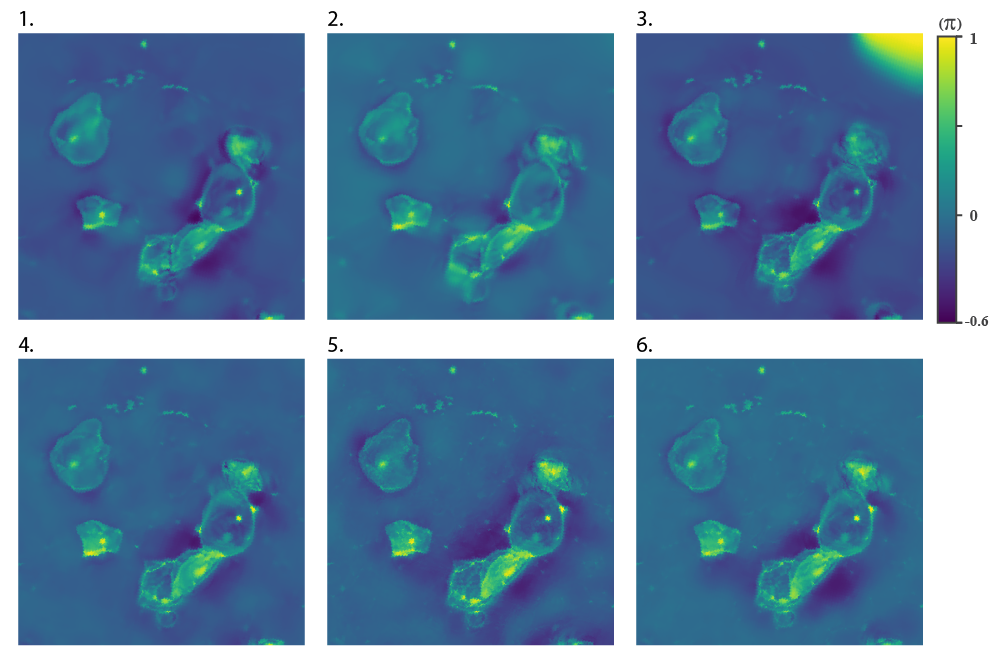


**Figure S2.** Output phase images reconstructed by different network architectures. The numberings correspond with the rows of Table S1.

**Table S1.** Six different network structures and their specifications. Each layer performs a 2-fold bilinear.

|  | Channels per layer, begins from the first convolution layer | Input tensor shape | Total parameters | Trainable parameters | Last convolution layer parameters |
| --- | --- | --- | --- | --- | --- |
| 1 | [128, 128, 128, 128, 128, 128] | 8×8×128 | 119,426 | 117,634 | 17,024 |
| 2 | [256, 256, 256, 256, 256, 256] | 8×8×256 | 468,226 | 464,642 | 57,816 |
| 3 | [512, 256, 128, 128, 128, 128] | 8×8×1024 | 382,210 | 380,162 | 17,024 |
| 4 | [128, 128, 128, 128, 128] | 16×16×128 | 102,402 | 100,866 | 17,024 |
| 5 | [256, 256, 256, 256, 256] | 16×16×256 | 401,410 | 398,338 | 57,816 |
| 6 | [512, 256, 128, 128, 128] | 16×16×1024 | 365,186 | 363,394 | 17,024 |

To clarify the influence of network design on reconstruction results of a problem, 6 different networks are defined which their properties are denoted in Table S1 and their outcomes are shown in Fig.S2. For each case, the model is optimized for 30000 iterations and all hyperparameters are similar.

As can be understood from the phase images in Fig.S2, networks with smaller input channel sizes (8×8) produce over-smoothed images with inaccurate low-frequency details. Furthermore, networks with fewer parameters favor reconstructing more significant structures. Although a pyramid structure of channel sizes is usually preferred due to its robustness (Table S1.3,6 and Fig.S2.3,6), Fig.S2.5 has better resolution. Since our priority is keeping the number of parameters lower than the number of data points, a network with similar layers having 256 channels per layer (Table S1.5, Fig.S2.5) is preferred. Figure S3 schematically shows the structure of the adopted neural network.


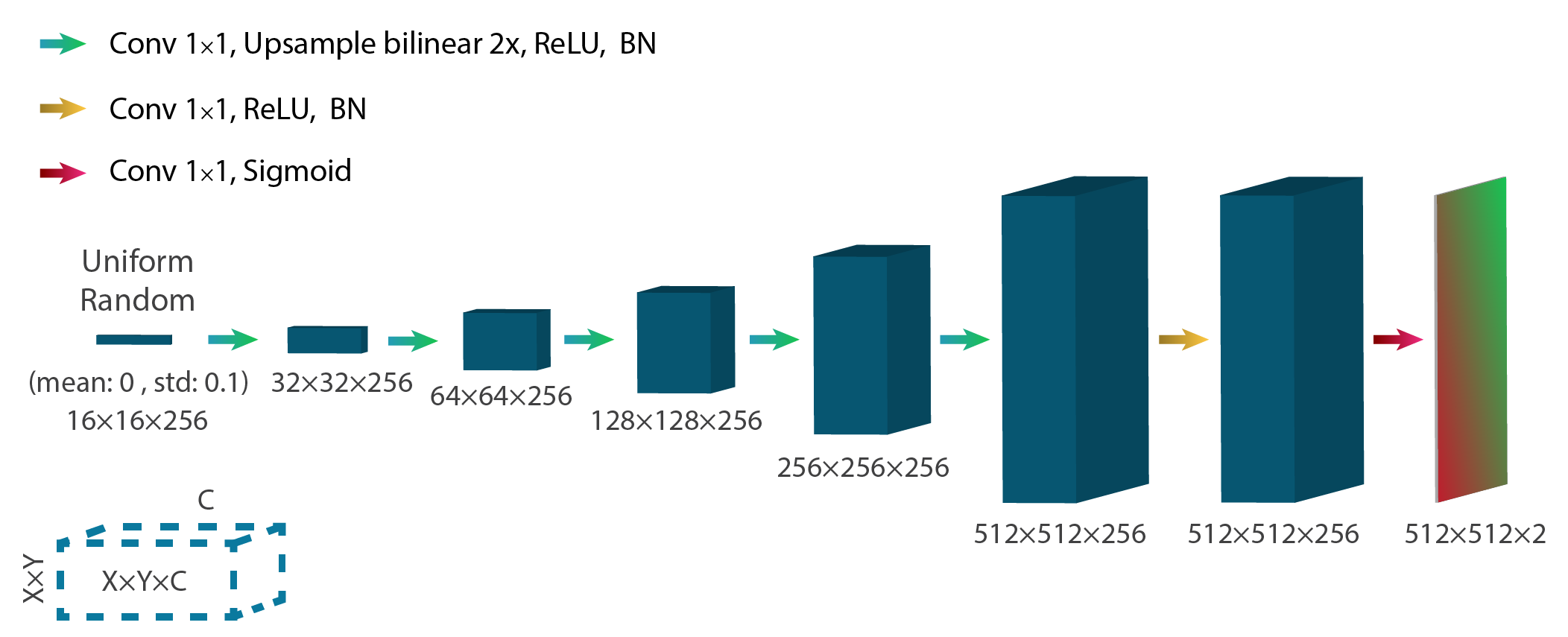


**Figure S3.** Structure of the employed deep decoder network.

1. H. Bay, A. Ess, T. Tuytelaars, and L. Van Gool, "SURF: Speeded-Up Robust Features," Comput. Vis. Image Underst. CVIU **110**, 346–359 (2008).
